# The impact of childhood RSV infection on children's and parents' quality of life: a prospective multicenter study in Spain

**DOI:** 10.1186/s12879-021-06629-z

**Published:** 2021-09-06

**Authors:** Eva Díez-Gandía, Carla Gómez-Álvarez, Mónica López-Lacort, Cintia Muñoz-Quiles, Isabel Úbeda-Sansano, Javier Díez-Domingo, Alejandro Orrico-Sánchez, Study collaborators, Fernando Calvo Rigual, Eva Suarez Vicent, Carmen Mañes, Elena Martí, Teresa Cerdán, Antonio Soriano Arandes, Lucia Losada Pavón, Airam Álvarez Sánchez, Gemma Ricós Furió, Tomás Pérez Porcuna, Noemí Magro Benito, Javier Martínez Díaz, Jessica Ortiz, Ana Mangas, Mónica García, Patricia Rovira, Marta Urgellés, Marta Pozuelo

**Affiliations:** 1 Hospital Lluís Alcanys, Xativa, Valencia, Spain; 2 Vaccine Research Department, Fundación Para el Fomento de La Investigación Sanitaria y Biomédica de la Comunitat Valenciana, FISABIO-Public Health, Valencia, Spain; 3 Health care center La Eliana, Valencia, Spain; 4 Universidad Católica de Valencia ‘San Vicente Mártir, Valencia, Spain

**Keywords:** Health‐related quality of life, Respiratory syncytial virus, Children, Quality‐adjusted life years, Respiratory disease

## Abstract

**Background:**

Several immunisation candidates against RSV are in late-stage clinical trials. To evaluate the benefits of a potential vaccination programme, both economic and health benefits will be needed. Health benefits are usually measured in Health-related Quality of Life (HRQoL) loss using standardised questionnaires. However, there are no RSV-specific questionnaires validated for children under 2 years, in whom most RSV episodes occur. Therefore, HRQoL estimates are taken from literature or inadequate tools. We determined HRQoL loss and direct costs due to an RSV episode in children younger than 2 years and their caregivers during a month of follow up, using a new questionnaire administered online.

**Methods:**

An observational prospective multicentre surveillance study was conducted in children aged younger than two years. Children were recruited from 8 primary care centres and 1 hospital in the Valencia region and Catalonia (Spain). RSV-positive cases were obtained by immunochromatographic test. HRQoL was assessed using a new ad-hoc 38 item-questionnaire developed. Parents of infected children completed 4 questionnaires at four timepoints (day 0, 7, 14 and 30) after diagnosis.

**Results:**

117 children were enrolled in the study and 86 (73.5%) were RSV + . Median (interquartile range; IQR) scores were 0.52 (0.42–0.68), 0.65 (0.49–0.79), 0.82 (0.68–0.97) and 0.94 (0.81–1), for days 0, 7, 14 and 30, respectively. Compared to total recovery (Q30), HRQoL loss was 37.5%, 31.5% and 8.9% on days 0, 7 and 14 since diagnosis of the disease. The total median cost per patient (including treatments) was €598.8 (IQR: 359.63–2425.85).

**Conclusions:**

RSV had almost 40% impact on HRQoL during the first week since onset of symptoms and the median cost per episode and patient was about €600. These results represent a substantial input for health-economic evaluations of future RSV-related interventions such as vaccination.

**Supplementary Information:**

The online version contains supplementary material available at 10.1186/s12879-021-06629-z.

## Background

Respiratory syncytial virus (RSV) is a major cause of acute lower respiratory infection (ALRI) in young children, especially in children < 2 years. Nearly all children get an RSV infection at least once by the time they are two years’ old. Globally, the RSV burden is huge, accounting for about 33 million ALRI episodes, 3 million hospital admissions and 60,000 in-hospital deaths [1, 2].

At present, no vaccine is available for RSV prevention. Although 6 monoclonal antibodies and more than 40 RSV vaccine candidates are under study, 18 of them in clinical trials [3]. Some have recently shown their efficacy in phase III studies [4, 5]. Therefore, a preventive intervention for RSV is on the horizon and could be available soon.

Decision-makers, health managers and health professionals will need economic evaluations of the different immunisation strategies before their licensing. A cornerstone of such economic evaluationsis the cost-utility analysis (CUA) [6, 7]. In addition to costs, crucial to CUA calculation is an assessment of how the disease affects health-related quality of life (HRQoL). When expressed over time as Quality Adjusted Life Years (QALYs) this permits standardised comparisons between different healthcare interventions (i.e. immunisation strategies). Therefore RSV-related costs and impact of HRQoL are two of the pillars of the CUA of RSV immunisation strategies.

However, validated RSV-related HRQoL and QALY loss for very young children has not yet been published [8, 9]. One of the reasons of this scarcity is the lack of adequate tools to estimate HRQoL of children between 0–2 years of age in whom most RSV episodes occur. Impact of HRQoL can be evaluated using multi-attribute questionnaires [10]. EQ‐5D [11], HUI [12] and SF-36 [13] are validated questionnaires most frequently used to elicit HRQoL. However, these questionnaires have been validated for children above the age of 5 years [14–16]. Other questionnaires available for youngest children [17–20] are limited from 2 months old and do not include RSV-related questions. In addition, they do not provide a single score capturing overall HRQoL. Moreover, HRQoL loss of the parents or caregivers of infected children, which proved to be significant for rotavirus [21–23], are not usually included in the questionnaires.

The only publication identified determining QALY loss due to an RSV episode in individuals < 5 years old [24] is based on model predictions from older children (5–14 years old), instead of using an age-adapted questionnaire.

Similarly, the few CUA of the RSV immunisation strategies published [25–28] did not estimated RSV-related QALY loss in young children for their analysis. Instead, they used non-RSV related utilities derived from literature. One of the most commonly used studies is the one describing the utility of 5 year-old preterm children with a history of chronic lung disease, where participants completed the questionnaires 5 weeks after their symptoms [21]. This fact might generate biased data that can lead to erroneous decisions in health policy.

In this study, we determined the HRQoL loss and direct costs due to an RSV episode in children under 2 years’ old, positive for RSV, and their caregivers during a month of follow up, using a new questionnaire administered online. This article also explains how HRQoL scores were derived from the 5 dimensions of the questionnaire. These are crucial estimations for CUA of the RSV immunisation strategies coming and would support health policy-makers’ decisions.

## Methods

### Study design and population

An observational prospective multicentre surveillance study was conducted in children aged less than two years during the 2018‐19 RSV season (October – May). A total of 20 paediatritians belonging to 8 public primary care centres in Spain (4 in the Valencia region and 4 in Catalonia) and 1 public hospital in the Valencia region participated in the study. All minors belonging to the quota of pediatricians who met the inclusion criteria were offered to participate in the study.

Inclusion criteria: (1) children under 24 months of age assisted in the participating centres, (2) suspected RSV infection or ALRI, (3) fewer than 7 days since the onset of symptoms. Recurrent RSV infections were also considered.

Exclusion criteria: (1) children that received palivizumab 40 days prior to the visit, or (2) intravenous immunoglobulin 30 days before, (3) or those whose parents/caregivers had difficulty understanding or communicating in the Spanish language.

### RSV diagnosis

A nasopharyngeal swab was obtained from all consented participants. A one-step immunochromatographic test (OPERON ®) was used for the qualitative detection of RSV. The sensitivity and specificity of the test was 96.4% and 97.6%, respectively [29].

### eCRF: electronic case report form

Children’s clinical data, including general condition, axillary temperature, acyanosis and modified Wood-Downes score [30, 31] (oxygen saturation, breathing rate, heart rate, wheezing, crackling and accessory musculature). Other additional variables were also collected, such as: comorbidities, prematurity, vaccination history, sociodemographic information, exposure to tobacco smoke at home, breastfeeding, nursery school, inclusion date, immunodeficiency (See Additional file 1: Annex S1: eCRF).

### Follow-up

Parents/caregivers of the RSV-confirmed subjects who satisfied the inclusion criteria were asked to fill out an online quality of life questionnaire on days 0 (Q0; recruitment day), 7, 14 (Q7, Q14; follow-up evaluation of the disease) and 30 (Q30; considered as complete recovery, which served as intra-subject control). Participants received the questionnaires by email on the days they had to fill them out. Permission was also requested to call them by phone.

### Questionnaire information

An ad hoc questionnaire was developed for the study, in collaboration with experts (paediatricians) and patients’ associations. It consisted of a 38-items questionnaire adapted to the disease and the parent’s age group. The questionnaire was built taking as a reference another questionnaire [23] validated for rotavirus gastroenteritis where our group participated. Dimensions used and the inclusion of parent’s related questions were considered useful for the RSV questionnaire. Questions were divided into five dimensions which gathered information regarding the child’s symptoms and behaviour (including number of days), parent’s concerns, emotions and the impact of the infection on family activities. See Additional file 1: Annex S2 for the full questionnaire. Additionally, families were also asked to complete information related to the healthcare resource consumption: visits to the paediatrician, emergency rooms, hospitalisations and Intensive Care Unit (ICU) stays. RSV-related treatments were also collected (see Additional file 1: Annex S3).

### PDA/Tablet version questionnaire

In order to avoid loss of adherence in the return of questionnaires, the survey was developed through the Google Forms ® platform (fully adaptable to tablets, computers or smartphones). A link to the questionnaire was sent by email to the parents/caregivers of the subjects every day that they had to complete it. For those few cases with no Internet access or email account, phone calls were also possible. Time spent completing the questionnaire was estimated at 5.5 min.

### Healthcare resource consumption and related costs

Healthcare costs estimation was estimated per RSV + episode. The use of the following resources was studied: primary care visits, emergency visits (at primary care and hospital setting), hospital stays, ICU stays and RSV-related treatments (paracetamol, ibuprophen, salbutamol, oral steroids, physiological serum, inhalation mask and chamber, budesonide and antibiotics). Although the use of antibiotics is not appropiate, it is sometimes wrongly prescribed in Spain and we decided to include it for the resources estimations. This information was gathered in Q0, Q7, Q14, Q30.

### Statistical analysis

Sociodemographic and clinical data were summarised using frequencies and proportions by RSV + and RSV-. RSV infection risk was analysed by a multivariable logistic regression adjusted by age, historical bronchiolitis, and caregivers smoking habit.

*QoL weights:* questionnaires item responses on day 0, 7, 14 and 30 were summarised by frequency and proportion. For the quality of life analysis of each questionnaire, item responses were scored using the following algorithm (unweighted method): (1) Each response item was assigned a value according to its gravity (1 = best level, 5 or 9 = worst level). (2) scores per response and child were summed. (3) The sum of the scores (in overall questionnaire and by dimension) were standardised using the min–max normalisation to a 0–1-point scale, with 1 indicating the best quality of life and 0 the worst:

$$U_{i} = \, \frac{{P_{i} - r_{\min } }}{{1 - r_{\min } - r_{\min } }}, 0 \le U_{i} \le { 1}$$

where *U*_*i*_, is the value of the HQoL for the children i, *P*_*i*_ is the sum of the scores per response, and *r*_min_ and *r*_max_ the minimum and maximum possible attributable score.

Quality of life was summarised by mean, median, range, and interquartile range.

*HRQoL loss*: loss of quality of life was estimated in general and by age group comparing the average quality of life during the course of the disease, with optimal health (30 days after onset of symptoms), using a mixed effect log-normal model including the subject as random effect.

Descriptive analysis of helthcare resouce utilization and their related costs were performed overall and by resource unit, including means, medians, IQR, and standard deviations. The costs per children were calculated as the number of resource units utilized multiplied by the costs per resource unit. Prices were estimated from the regional rates law [32] and treatments costs from the Bot-Plus database [33]. Prices used are presented in Additional file 1: Annex S6.

## Results

### Demographic and clinical characteristics of the study population

A total of 117 children were enrolled in the study, of those 38 required hospitalisation. There were 86 (73.5%) children RSV + (33 hospitalized). Sociodemographic and clinical characteristics of the population are presented in Table 1. Half of the enrolled subjects (53.8%) were male. RSV + children were younger and with more siblings. All preterm children enrolled were RSV + . Clinical conditions were different depending on the virus causing the disease. Conditions such as fever, hypoxemia, wheezing and retractions were higher within the RSV + patients. 5 of the patients enrolled in the study presented cyanosis and they were all RSV positive.

**Table 1:** Sociodemographic and clinical characteristics of the enrolled subjects

		RSV	
		Negative (%)	Positive (%)
N	117	31 (26.5)	86 (73.5)
		RSV-n = 31 (%)	RSV + n = 86(%)
Gender	Boys	17 (54.8)	46 (53.5)
	Girls	14 (45.2)	40 (46.5)
Age in months	0–5	15 (48.4)	56 (65.1)
	6–11	7 (22.6)	20 (23.3)
	12–23	9 (29)	10 (11.6)
Siblings	Yes	19 (61.3)	64 (74.4)
	No	12 (38.7)	22 (25.6)
Breastfeeding	Yes	24 (77.4)	61 (70.9)
	No	7 (22.6)	25 (29.1)
Preterm*	Yes	0 (0)	6 (7)
	No	31 (100)	80 (93)
Family smoker	Yes	6 (19.4)	36 (41.9)
	No	25 (80.6)	50 (58.1)
General condition	Good	28 (90.3)	65 (75.6)
	Irritability	2 (6.5)	16 (18.6)
	Lethargy	1 (3.2)	5 (5.8)
Cyanosis	Yes	0 (0)	5 (5.8)
	No	31 (100)	81 (94.2)
Fever** (ºC)	Yes	4 (12.9)	13 (15.1)
	No	27 (87.1)	73 (84.9)
Heart rate (bpm)	< 100	1 (3.2)	3 (3.5)
	100–139	12 (38.7)	24 (27.9)
	140–169	8 (25.8)	40 (46.5)
	> 170	5 (16.1)	9 (10.5)
	NA	5 (16.1)	10 (11.6)
Crackling	Yes	12 (38.7)	50 (58.1)
	No	19 (61.3)	36 (41.9)
Modified Downes Score			
O_2_ saturation (%)	< 90	0 (0)	2 (2.3)
	90–94	2 (6.5)	7 (8.1)
	95–100	24 (77.4)	69 (80.2)
	NA	5 (16.1)	8 (9.3)
Respiratory rate (rpm)	20–44	17 (54.8)	32 (37.2)
	45–65	12 (38.7)	33 (38.4)
	> 65	2 (6.5)	10 (11.6)
	NA	0 (0)	11 (12.8)
Wheezing**	Slight	21 (67.7)	55 (63.9)
	All expiration	8 (25.8)	20 (23.3)
	Both	0 (0)	7 (8.1)
	NA	2 (6.5)	4 (4.7)
Accessory muscles used***	Mild	29 (93.5)	75 (87.2)
	Moderate Intense NA	2 (6.5) 0 (0) 0 (0)	11 (12.8) 0 (0) 0 (0)

*Preterm: < 37 weeks. **Considered axillar fever from 37.9; **Wheezing: both means inspiratory and expiratory audible without stethoscope; ***NA means no data, mild means none or mild intercostal, moderate means intercostal moderate or suprasternal, intense means wobble or flutter

### Risk factors

Abovementioned clinical and sociodemographic characteristics were considered as a potential risk factors in the model. Differences found between RSV positives and negatives were explained by smoking habit and previous history of bronchiolitis. Children exposed to smoke at home increased their risk of RSV by 3 times (OR = 3.01 (1.16–8.95)). On the contrary, previous history of bronchiolitis was related with less probability of RSV infection (OR = 0.09 (0.02–0.4)). Age was included as a confounding factor.

### Questionnaire responses

**Fig. 1:**
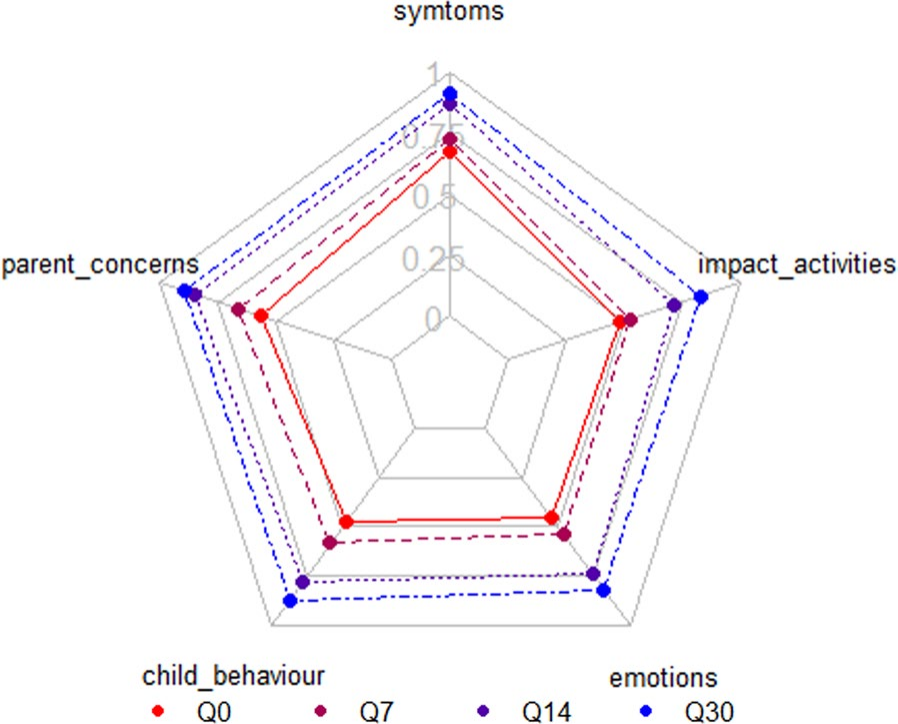
Radar chart with the five domains of the questionnaire. This graph shows the evolution of the scores in each domain for all subjects 1 (Q0), 2 (Q7), 3 (Q14) and 4 (Q30) weeks after RSV diagnosis

Questionnaires were fully completed by 77 (4 parents only responded 3 and 5 parents only one) of the 86 RSV + children’s relatives included in the study. Radar chart with the average scores of the 5 domains (children’s symptoms, parent’s concerns, impact on parent’s activities, children’s behaviour and parents’ emotions) of the questionnaire is presented in Fig. 1. Overall, the disease became milder as time passed and the score improved correlatively over time. Scores by dimension are presented in Additional file 1: Annex S4.

**Fig. 2:**
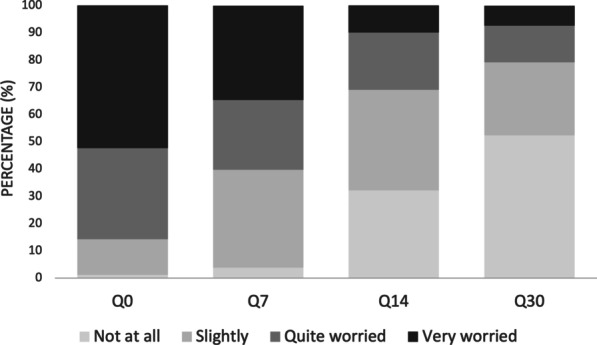
Global parents’ concern responses about the child’s illness in the four questionnaires. Q0, Q7, Q14 and Q30 correspond to questionnaires after 0, 7, 14 and 30 days since onset of symptoms, respectively

Parental concern about the child’s illness decreased over time and is presented in Fig. 2. 88% of parents were very and quite worried after the first week since diagnosis (Q0). The percentage of parents quite or very worried dropped to less than 20% in the last week (Q30). Ratings by questionnaire dimension are presented in Additional file 1: Annex S2.

Of the 325 questionnaires completed, 96 (29.5%), 62 (19.1%) and 167(51.4%) were completed within 24 h, 24-48 h and after 48 h since the email with te questionnaire was sent, respectivelly.

### Health-related quality of life impact

#### Questionnaire-derived HRQoL weights

**Fig. 3:**
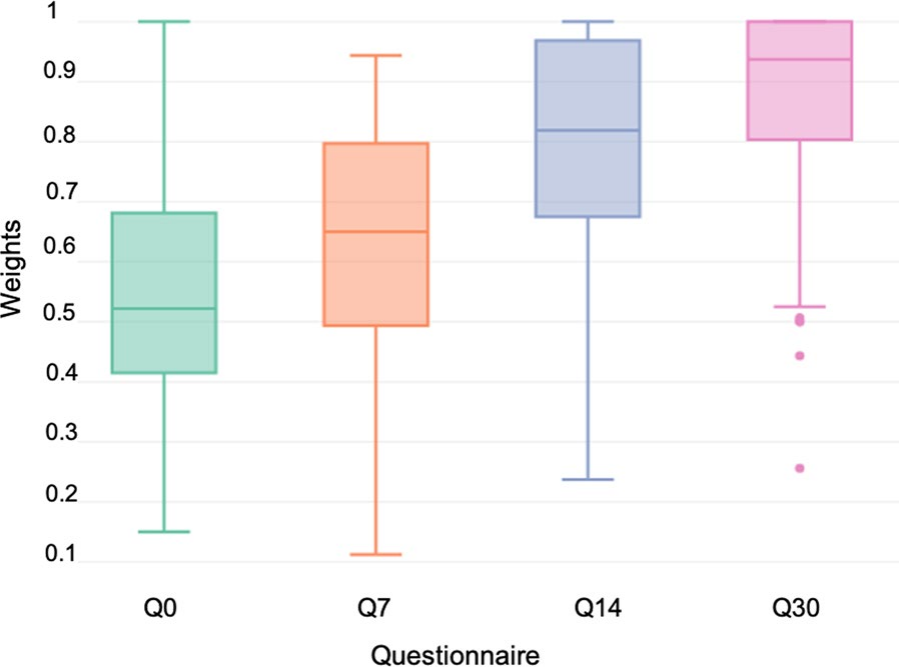
Questionnaire-derived weights for days 0, 7, 14 and 30 since diagnosis of RSV. Q0, Q7, Q14 and Q30 correspond to questionnaires at day 0, 7, 14 and 30, respectively

Variations in the HRQoL scores during the month of follow-up are presented in Fig. 3. Median (IQR) scores for Q0, Q7, Q14 and Q30 were 0.52 (0.42–0.68), 0.65 (0.49–0.79), 0.82 (0.68–0.97) and 0.94 (0.81–1), respectively. Scores improved as time since onset of symptoms increased (Fig. 3).

Additionally, scores by age group and dimension are presented in Additional file 1: Annex S5. No differences between age groups were found.

#### Loss of HRQoL

Overall, the loss of HRQoL in RSV-infected children and their relatives was between 9 and 38% for 3 weeks after infection. Compared to the total recovery (Q30), HRQoL loss was 37.5%, 31.5% and 8.9% in days 0, 7 and 14 since the diagnosis of the disease (Table 2). No differences were found between the different age groups or sex.

**Table 2:** Loss of quality of life of days 0 (Q0), 7 (Q7) and 14 (Q14) compared to day 30 (considered as complete recovery). IQR means interquartile range

Loss of Health-related quality of life				
Median (IQR)				
		Q0	Q7	Q14
Total		37.5 (33–41.7)	31.5 (26.5–36.1)	8.9 (2.3–15)
Age	0–5 months	35.8 (30.2–40.9)	31.5 (25.4–37.1)	7.5 (− 0.6–14.9)
	6–11 months	39.6 (28.7–48.8)	31.7 (19.3–42.1)	8 (− 8.6–22)
	12–23 months	43.2 (31.9–52.6)	31.4 (17.2–43.1)	17.8 (1.5–31.4)
Sex	Male	38.2 (32–43.8)	28.1 (20.9–34.7)	4.7 (− 4.8–13.4)
	Female	36.6 (29.9–42.6)	35.5 (28.5–41.8)	13.7 (4.5–22)

### Healthcare consumption

Of the 86 RSV episodes studied, 82 (95.4%), 52 (60.5%), 53 (61.6%), 30 (34.9%) and 2 (2.3%) required paediatrician visits (P), Emergency Department visits at Primary Care (EDPC), Emergency Department visits at hospital (EDH), hospital stay and ICU stay, respectively. Average visits per patient and resource unit are summarized in Table 3. The median number of visits (per episode) to the paediatritians, the EDPC and the EDH was 4.94, 1.85 and 1.62 times, respectively. The median number of hospital stays and ICU was 1.62 and 0.06, respectively.

**Table 3:** Healthcare consumption. P (Paediatrician), EDPC (Emergency Department at Primary Care), EDH (Emergency Department at hospital) and ICU (Intensive Care Unit); HC (Healthcare consumption)

Setting	Average per patient (Median;IQR)	HC Total	Costs per visit (€)	Average costs (€) per children (Median;IQR)	Total costs (€)
P visits	4.94 (4.0; 3–6)	420	42.57	208.4 (170.28;127.71–255.42)	17,921.97
EDPC visits	1.85 (1.0; 0–2)	157	155.89	155.89 (286.4; 0.0–311.78)	24,630.62
Hospitalisations	2.35 (0.0; 0–3)	200	544.08	1280.0 (0; 0.0–1632.0)	108,816.00
EDH visits	1.62 (1.0; 0–2)	138	155.89	155.89 (250.15; 0.0–311.78)	21,512.82
ICU stays	0.06 (0.0; 0–0)	5	1365.29	512.4 (0;0–0)	6826.45
Specialists visit	1.29 (0.0; 0–1)	111	107.33	138.53 (0; 0–107.33)	11,913.63

Regarding the pharmaceutical treatment used, paracetamol and salbutamol were used in more than 60% of the patients, followed by ibuprofen, oral steroid and antibiotics in more than 20% of the patients. Physiological serum was used in almost all children (93%) (see Additional file 1 for details).

Overall, the total median cost per RSV episode (including treatments) was €713.45 (IQR: 397.11–2370.0). The overall cost for al RSV studied episodes was 193.410,07€.

## Discussion

This is the first study estimating the HRQoL impact of RSV using questionnaire-derived weights from RSV-confirmed children under 2 years of age and their relatives during the course of infection. A new ad hoc RSV-specific questionnaire has been developed and used for assessing HRQoL in very young infants infected and their parents. Additionally, the direct costs associated with RSV infection during one month of follow-up were also estimated in the same cohort of participants.

Comparing it to a baseline reported HRQoL at day 30 since onset of symptoms (Q30; baseline), we found that RSV was associated with a mean HRQoL loss in children and their parents of 38%, 32% and 9% during the first, second and third week after the diagnosis, respectively. These results are lower than the only published study estimating a model-estimated HRQoL loss of 0.54 (95% CI 0.14–0.95) in children under 5 years [24]. However, their estimates were not obtained directly, instead they used the responses from the older suspected cases. Using these responses they built a regression model to predict the (model‐estimated) HRQoL loss. The choice of respondents may have a substantial influence on such valuations [34]. Some authors consider patients to be the most appropriate judge of his/her health experience [35]. Ideally, a survey, which asks about the patient’s current health, should be taken repeatedly during the period of illness, in order to estimate the loss in health-related QoL. In addition, they used the EQ-5D-3L questionnaire, where questions are not related with the disease of study and all questionnaires were completed some weeks after symptoms. Although it is a good approximation, all of these factors can contribute to potentially biased results.

Although it cannot be directly comparable, our results are aligned with another study estimating HRQoL due to RSV infection in children under 5 years [9]. They found a disutility range from 0.16 to 0.62 using a time trade‐off method in which QoL loss was estimated by asking participants about a hypothetical illness that they had not experienced. But these methods are susceptible to several biases [36]. In addition, our results could be also comparable to utilities found in other respiratory diseases. The median health utility of children 0–4 years of age during the influenza episode was around 0.64 [37]. Therefore, the impact on HRQoL is around 36%. However, mean HRQoL loss of child and related adults with rotavirus gastroenteritis (0.68–0.74) seems lower [21].

Our results have been estimated using a new ad hoc RSV-specific questionnaire, because there were no other questionnaires available specific to RSV for children between 0–2 years of age. The most frequently used questionnaires, such as EQ-5D, HUI or SF-36 are not validated for children under 5 years old [14–16]. Other questionnaires were indicated for children under 5 [38], but not for under 2 years. Two other questionnaires useful for under 2 years [39, 40] were not validated for children under 2 months. However, the peak incidence of RSV is before 2 months [41] and half of our children were younger than 2 months. Therefore, in collaboration with paediatricians’ and patients' associations, we developed a 38-item questionnaire adapted to the disease and the patient’s age group (0–2 years). Importantly, it also gathers information about the illness’s impact on parents/caregivers. Parents of infected children may experience worry, anxiety and distress. Moreover, family’s activities may also be disrupted (i.e. work responsibilities, housekeeping or leisure). So information about how the illness impacts the family should be also included for a fuller understanding of the burden of RSV disease. To do this, we relied on the previously validated questionnaire used to estimate the impact of gastroenteritis due to rotavirus in infants [23]. Nevertheless, the convergent and discriminant capacity of the questionnaire is being evaluated in a new study. This study will also allow us to transform the scores to an anchor-based range (from 0 = death to 1 = full health). After this transformation, we will be able to estimate QALYs that are necessary for economic evaluations of future immunisation strategies.

We estimated QoL loss using the last reported questionnaire (Q30) as baseline, i.e. we assumed that by the end of the study individuals had returned to normal health since the duration of RSV episodes is around 28 days [42]. Parents’ general health perception varies depending on their personal situation (i.e. number of children, employment and psychological situation, their own health and stress, etc.) [43]. Therefore, one of the most appropriate ways to measure the impact on quality is by comparing it with the baseline quality of life of the same subjects. We expected to find differences in HRQoL according to the different age groups studied. But the low number of subjects included may have influenced this. Moreover, the fact that the QoL found in our study improved over time (from day 0 to day 30), might be significant for the validity of our questionnaire.

Regarding risk factors, it was observed that children under two years of age exposed to smoke were three times more likely to have a positive episode of RSV. This was also analysed in a study carried out in Vienna, where recent exposure to tobacco smoke was associated with greater severity of symptoms and lower oxygen saturation [44]. Other studies in premature infants found that this was also a risk factor [45]. Patients that had an infection with RSV were younger, had more siblings and were breastfed less, which is consistent with literature as they are well known risk factors, as well as prematurity, for presenting RSV ALRI. All preterm children enrolled in our study were RSV positive. Unfortunately, the sample size was small and we did not have enough statistical power to infer conclusions. But age was a modifying effect for other risk factors such as smoking, which increased the relative risk in children younger than 6 months of age. There was no relationship between nursery school attendance or vaccination status with having RSV. A new study with a larger sample size is underway to evaluate these factors.

Overall, RSV + patients had the worst general condition when they were included in the study. This could be explained by the fact that RSV can cause a more severe ALRI than other respiratory viruses or because the patients in this group were younger. Interestingly, we observed that having a previous episode of bronchiolitis could influence the result of being RSV + . These children were approximately 90% less likely to have a positive RSV. This could be explained by the observation that children with a previous RSV infection have a higher change of developing bronchiolitis [46]. Future immunisation strategies may have a great impact not only on QoL, but also on recurrent bronchiolitis.

Direct RSV-related healthcare consumption, including treatments, was also estimated. Overall, RSV-infected patients visited the paediatrician and emergency rooms a median of 4 times and 1 time per episode, respectively. 5.5% of RSV-infected children were hospitalised and 3 needed ICU admission. These estimates are aligned with previous population-based RSV-hospitalisations estimates of around 7.5% in the same region [41]. The median cost per RSV episode was around €600 (IQR: 359.63-2425.85). These estimates included both inpatients and outpatients. These costs would be assumed by the Spanish National Health System (SNHS) that leads nearly universal coverage of all citizens, providing care based on need and free at the point of delivery, except for a cost-sharing scheme for pharmaceuticals dispensed out of hospitals [47]. A recent review published the overall costs of RSV episodes in both inpatients and outpatients in several countries [48]. They estimated an average cost per episode of €3,452 and €299 for inpatients and outpatients, respectively. The differences found could be related to the fact that most of our participants were outpatients. Interestingly, even though no pharmacologic treatment has shown an effect on bronchiolitis, inhalators, steroids and antibiotics are still commonly prescribed, with salbutamol being the most prescribed. Very few studies investigated the indirect costs incurred by parents or families taking care of children with RSV infection [48]. The new version of the questionnaire used in this study will include questions to address this gap.

The limitations of the study were as follows: first, since we developed a new questionnaire, it has not been validated in the different age populations or in asymptomatic children. Second, the delay in answering the questionnaires. About 50% of questionnaires were completed at least 48 h after the scheduled date, so they may be subject to recall bias. Third, the study is only representative of children whose parents sought medical attention. It’s striking that in children under 6 months of age, the loss of QoL and the symptoms score was less than for the older age groups. This could also be justified by the sample distribution, as the number of children below 6 months is higher. On the other hand, parents may have more trouble interpreting the youngest child’s symptoms [49]. In addition, maternity and paternity leave might have also influenced this. The impact of children’s illness during maternity leave could have been lower than during the working day. Therefore, the youngest cases might have coincided with maternity and paternity leave. Finally, for the healthcare consumption, only one box was taken into account per treatment used and per episode. If more boxes were used, they were not considered.

## Conclusions

There is still a gap in the analysis of the RSV QALYs in young children. This study is the first one attempting to measure the HRQoL of RSV patients under 2 years of age, where the illness is most severe. RSV had almost 40% impact on HRQoL during the first week since onset of symptoms and the median cost per episode and patient was about €600. These results are the basis for correctly estimating the QALYs and will be of significant interest to policy-makers, government decision-makers, pharmaceutical research, and healthcare practitioners, since they represent substantial input for cost-utility evaluations of the future RSV-related interventions such as vaccination.

## Supplementary Information

**Additional file 1:Annex S1.** Electronic case report form (eCRF). **Annex S2. **Questionnaire used for the study (english version). **Annex S3. ** Resource consumption—related questions. **Annex S4. **Questionnaire ratings by dimension. **Annex S5. **HRQoL scores by age group and dimension. **Annex S6**. Prices used in the healthcare resource consumption estimations.

## Data Availability

Additional analysis and results are available in the following dashboard (https://rotapp.shinyapps.io/appcv/). All statistical analyses conducted for this study are completely reproducible. The datasets used and/or analysed during the current study are available from the corresponding author on reasonable request.

## Abbreviations

RSV: Respiratory Syntycial Virus
QALYs: Quality Adjusted Life Years
HRQoL: Health Related Quality of Life
QoL: Quality of life
ALRI: Acute Low Respiratory Infections
IQR: InterQuartil Rate
Q0: Questionnaire of day 0 (day of recruitment)
Q7: Questionnaire of day 7
Q14: Questionnaire of day 14
Q30: Questionnaire of day 30 (recovered
