# Supplementary material for: The impact of childhood RSV infection on children's and parents' quality of life: a prospective multicenter study in Spain
Source: BMC Infect Dis. 2021 Sep 6;21:924. doi: 10.1186/s12879-021-06629-z (PMC8422742; doi:10.1186/s12879-021-06629-z)
Supplement: Supplementary file 1 — Additional file 1:Annex S1. Electronic case report form (eCRF). Annex S2. Questionnaire used for the study (english version). Annex S3. Resource consumption—related questions. Annex S4. Questionnaire ratings by dimension. Annex S5. HRQoL scores by age group and dimension. Annex S6. Prices used in the healthcare resource consumption estimations. [file 12879_2021_6629_MOESM1_ESM.docx]

**Annex 1: ELECTRONIC CASE REPORT FORM (eCRF)**

**Investigator identification data**

- Centre:
- Investigator’s name:

**Patient identification data**

- Date (dd/mm/yyyy):
- Subject number (last name of the investigator + consecutive ordinal numbers)
- Number of days since the onset of symptoms:
- Date of birth (dd/mm/yyyy):
- Sex (M/F):
- Number of siblings:
- Position among his/her siblings (ordinal: first…)
- Prematurity: No/Yes (gestational age (weeks)
- Breastfeeding (No/Yes: number of months ( )):
- Nursery (Yes/No)
- If a smoker: Number of cigarettes/day ( ) (father). Number of cigarettes/day ( ) (mother). Number of cigarettes/day ( ) (caregiver)
- Any smokers at home (Yes/No)
- Employment status:
  - Mother: Active, unemployed, inactive *
  - Father: Active, unemployed, inactive *

(*) Inactive people are retired people, people who cannot work because they are permanently disabled, or people who do not work because they engage in other unpaid tasks such as caring for the home, caring for sick relatives, etc.

- Educational level
  - Mother: no studies, primary, school graduate, technical, higher, postgraduate.
  - Father: no studies, primary, school graduate, technical, higher, NA.
- Email
- Telephone contacts:
- Person responsible for filling in the questionnaires (father/mother/caregiver)
- Post Code:

**Eligibility**

- Meets the inclusion criteria? (Yes/No/NA)
- Has signed the informed consent? (Yes/No/NA)
- Symptom onset date (dd/mmm/yyyy):

**Physical exam**

- General condition (good, irritability, lethargy):
- Cyanosis (Yes/No):
- Axillary temperature (XX, X ºC):
- Modified Wood-Downes score:
  - Oxygen saturation (XX air):
  - Respiratory rate (Number of breaths per minute):
  - Expiratory wheezing (mild / all expiration / inspiratory and expiratory audible without phonendoscope).
  - Accessory musculature (None, subcostal or mild intercostal / Intercostal moderate and suprasternal / Intense, wobble, flutter)
  - Heart rate (Not available / beats/min ( )

**Comorbidities**

- History of bronchial hyperresponsiveness: (Yes/No/Unknown)
- History of pneumonia: (Yes/No/Unknown)
- History of bronchiolitis: (Yes/No/Unknown)
- Other notable history of respiratory pathology: (Yes (which) /No/Unknown)
- Other non-respiratory antecedents of interest (cardiac, neuromuscular pathology, immunodeficiencies): Yes (which) /No/Unknown

**Vaccination history**

Indicate if any of the following vaccines are missing:

- Diphtheria, tetanus, pertussis: (Yes/No)
- Haemophilus influenza b: (Yes/No)
- Pneumococcus: (Yes/No)
- Rotavirus: (Yes/No)
- Meningococcal b: (Yes/No)
- Flu: (Yes/No)

**ANNEX 2: QUESTIONNAIRE USED FOR THE STUDY (ENGLISH VERSION)**

## CHILD’S SYMPTOMS

ANSWER THE FOLLOWING QUESTIONS ABOUT YOUR CHILD’S ILLNESS

1. During the last week, how many days has your child presented the following symptoms?

|  | 0 | 1/2 | 1 | 2 | 3 | 4 | 5 | 6 | 7 |
| --- | --- | --- | --- | --- | --- | --- | --- | --- | --- |
| Cough |  |  |  |  |  |  |  |  |  |
| Dyspnea (fast breathing, intercostal retractions…) |  |  |  |  |  |  |  |  |  |
| Wheezing |  |  |  |  |  |  |  |  |  |
| Cyanosis (Blueness in face/lips) |  |  |  |  |  |  |  |  |  |
| Less appetite than usual |  |  |  |  |  |  |  |  |  |
| Full days without eating |  |  |  |  |  |  |  |  |  |
| Fever |  |  |  |  |  |  |  |  |  |

2. In comparison with the previous week, your child’s symptoms this week have been: much worst, somewhat worse, the same, somewhat better, much better.

## CONCERN ABOUT CHILD’S SYMPTOMS

3. How worried have you felt about the following symptoms:

* If in question 1 you marked “0 days”, please go directly to question 6.

|  | Not worried | Slightly worried | Quite worried | Very worried |
| --- | --- | --- | --- | --- |
| Cough |  |  |  |  |
| Dyspnea (fast breathing, intercostal retractions…) |  |  |  |  |
| Wheezing |  |  |  |  |
| Cyanosis (Blueness in face/lips) |  |  |  |  |
| Less appetite than usual |  |  |  |  |
| Full days without eating |  |  |  |  |
| Fever |  |  |  |  |

4. If your child has presented fever, what was the highest temperature that he/she presented? (Select from 37 to 42ºC)

5. Overall, during the last week, how worried have you felt about your child’s disease? Not worried, slightly worried, quite worried, very worried.

6. In comparison to the previous week, your concern about your child’s symptoms has been: much worst, somewhat worse, the same, somewhat better, much better.

## CHILD’S BEHAVIOR DURING THE ILLNESS

The following questions are about your child’s behavior during the illness.

Please select the box of the answer that best applies to your son/daughter’s case.

7. During the last week, your child:

|  | Never | Sometimes | Often | Always |
| --- | --- | --- | --- | --- |
| Has slept more than usual |  |  |  |  |
| Has slept less than usual |  |  |  |  |
| Has cried more than usual |  |  |  |  |
| Has been more irritable |  |  |  |  |
| Has had less desire to play |  |  |  |  |
| Has been exhausted |  |  |  |  |
| Has been less attentive |  |  |  |  |
| Has needed more comfort |  |  |  |  |

8. In comparison to the previous week, your child’s behavior has been: much worst, somewhat worse, the same, somewhat better, much better.

## CONCERN ABOUT CHILD’S ILLNESS

The following questions are about what you felt as a father/mother about your child’s disease.

Please select the box of the answer that best applies to your case.

9. During the last week, have you had the following feelings concerning your child’s illness?

|  | No at all | A little bit | A lot | A great deal |
| --- | --- | --- | --- | --- |
| Sadness to see my child being ill |  |  |  |  |
| Impotence |  |  |  |  |
| Mental exhaustion |  |  |  |  |
| Physical exhaustion |  |  |  |  |
| Guiltiness |  |  |  |  |
| Fed up with the situation |  |  |  |  |

10. In comparison to the previous week, your emotions about your son/daughter’s illness this week have been: much worst, somewhat worse, the same, somewhat better, much better.

## YOUR DAILY ACTIVITIES DURING YOUR CHILD’S DISEASE

How much has your child’s illness interfered in your daily activities?

Please select the box of the answer that best applies to your case.

11. During the last week and concerning your child’s disease:

|  | 0 | 1/2 | 1 | 2 | 3 | 4 | 5 | 6 | 7 |
| --- | --- | --- | --- | --- | --- | --- | --- | --- | --- |
| How many nights did the illness disrupted your sleep? |  |  |  |  |  |  |  |  |  |
| How many days did you have to dedicate exclusively to your child? |  |  |  |  |  |  |  |  |  |
| How many days did you have to ask for help to someone else (parents, friends, neighbors…)? |  |  |  |  |  |  |  |  |  |
| How many days couldn’t he/she attend nursery school, or you couldn’t leave him/her home with a babysitter? |  |  |  |  |  |  |  |  |  |

12. During the last week and concerning your child’s disease:

|  | No at all | A little bit | A lot | A great deal |
| --- | --- | --- | --- | --- |
| Have you lost sleep hours? |  |  |  |  |
| Your child’s illness limited your leisure time? |  |  |  |  |
| Your child’s illness limited the time for doing the groceries |  |  |  |  |
| Your child illness limited the time for doing house chores |  |  |  |  |

13. Who has completed this questionnaire? Mother, Father or tutor.

**ANNEX 3: RESOURCE CONSUMPTION – RELATED QUESTIONS.**

1. During the last 7 days and concerning your child’s disease:

| Nº of days: | 0 | 1 | 2 | 3 | 4 | 5 | 6 | 7 |
| --- | --- | --- | --- | --- | --- | --- | --- | --- |
| Visits to the GP |  |  |  |  |  |  |  |  |
| Visits to the specialist doctor |  |  |  |  |  |  |  |  |
| Visits to the outpatients emergency |  |  |  |  |  |  |  |  |
| Visits to the hospital A&E department |  |  |  |  |  |  |  |  |
| Did your child need hospitalization? |  |  |  |  |  |  |  |  |
| Did your child need intensive care (hospitalization in ICU)? |  |  |  |  |  |  |  |  |

2. Did your child need the following medicines? (Mark all of the medicines that have been used)

- Paracetamol
- Ibuprofen
- Inhaled salbutamol
- Inhaled budesonide
- Montelukast
- Oral steroids
- Antibiotics
- Inhalation chamber
- Nasal washes
- Ipratropium bromide (Atrovent)
- Mucolytics

3. During the last week, did you have to attend with your child to the primary health care center or hospital to treat another disease? YES/NO. If you answered yes, please specify which other diseases (only in questionnaires at day 7, 14 and 30)

*Questionnaire was used in Spanish. Translation to English has not been validated yet.

**Annex 4: Ratings by dimensions**

The first dimension collected in the questionnaire was children’s symptoms and included two questions: number of days that the child presented symptoms regarding the disease (cough, choking sensation, wheezing, peripheric cyanosis (face/lips), lack of appetite and fever) and parental concern about the symptoms (not at all, slightly, quite or very worried).

The most common RSV-related symptom was cough, presented more than 5 days in around 60% of children during the first two weeks. Wheezing, lack of appetite and fever were the second most characteristic symptoms presented. About 70% of subjects experienced them at least once during the first week. On the other hand, a very low percentage (10%) had cyanosis (SFigure 1).


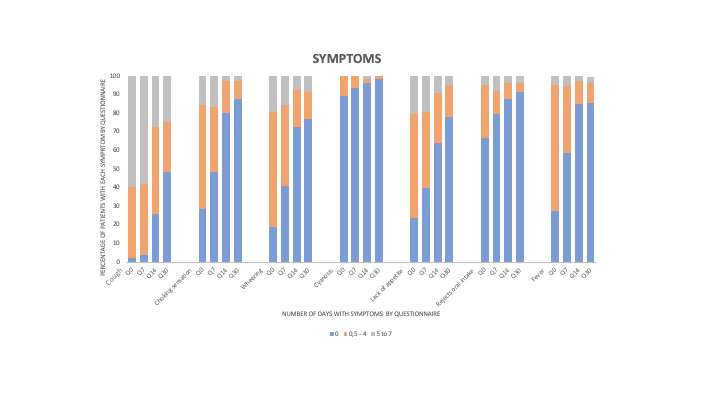


SFigure 1. Percentage of subjects presenting the studied symptoms over time (0, 0.5-4 or 5-7 days per week).

Relatives were mainly worried about cough, choking sensation and wheezing, with more than 60% of parents quite or very worried during the first week. 44% of parents were quite or very worried about fever.


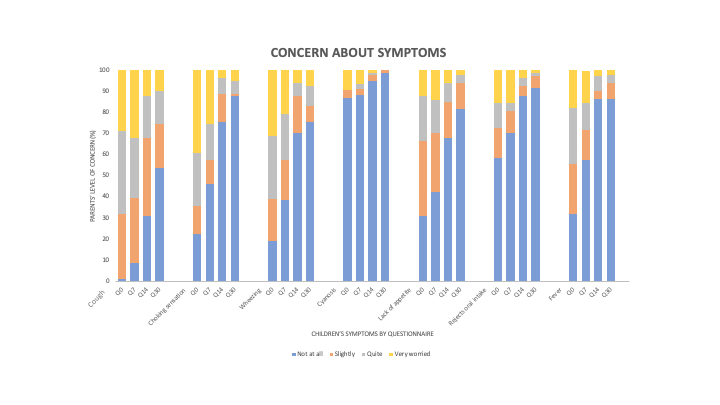


SFigure 2. Parents’ level of concern (%) about their children’s symptoms in the different questionnaires (Day 0, 7, 14 and 30).

The second dimension collected some children’s behaviours including sleepiness, irritability, playability, attention and comfort. Overall, around 70% of the children cried more and were irritable often/always during the first week. Around 50% were exhausted and played less than usual often/always during the first week (SFigure 3).


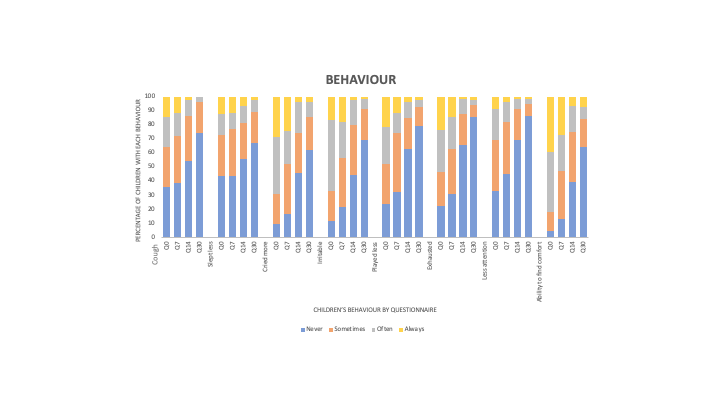


SFigure 3. Impact of illness on children’s behavior in the four questionnaires (day 0, 7, 14, 30).

The third dimension was presented in the main text and gathered the general level of parent’s concern about the child’s illness (not at all, slightly, quite a bit, or very worried) (Figure 1 of the manuscript).

The fourth dimension included parent’s feelings (sadness, impotence, mental and physical exhaustion and guilt) about the situation. The main feelings referred by the parents were sadness and helplessness (more than 70% of them felt them quite a lot/a lot during the first week). Mental and physical exhaustion were the second most common feelings (SFigure 5).


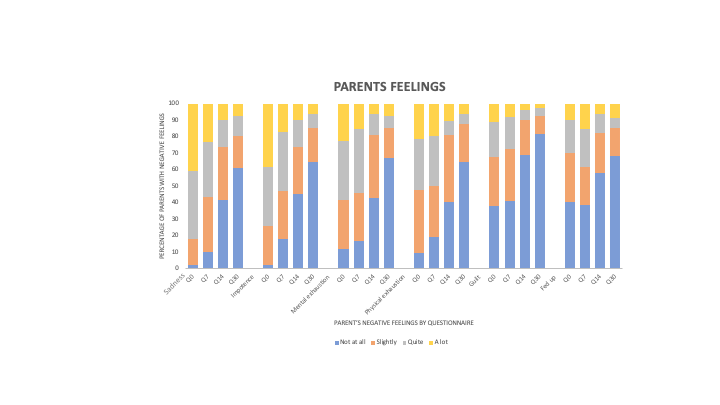


SFigure 5. Dimension 4. Level of parents’ negative feelings about the child’s illness (Sadness, impotence, mental and physical exhaustion, guilt or being fed up) in the four questionnaires (Day 0, 7, 14, 30).

Finally, the last dimension estimated the impact of the child’s illness on parent’s daily activities such as sleeping, independence, help needed (nursery or caregiver), leisure activities, shopping or household chores. RSV impacted parent’s sleeping and independence in 80% of cases during the first two weeks. They also needed help in more than 50% of cases during the first two weeks (SFigure 6). Leisure activities were affected quite a lot/a lot in around 50% of the families during the first two weeks (SFigure 7). From day 14 the impact on daily activities decreased, reaching no effect in the last days (SFigures 6 and 7).


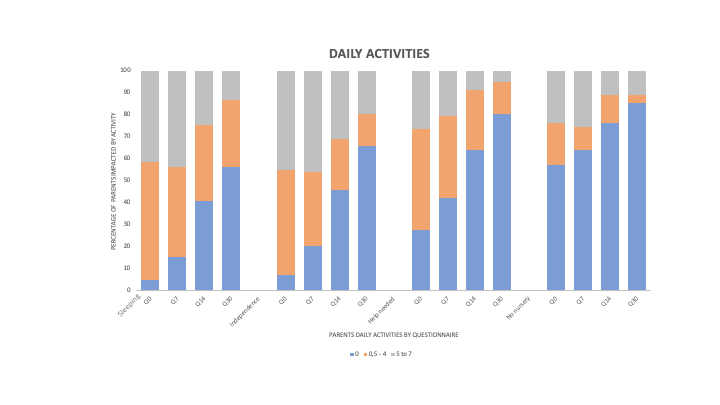


SFigure 6. Dimension 5. Impact of the child’s illness on parent’s daily activities such as sleeping, independence, help needed or not attending nursery, counted as days that the event occurred in the last week (0 days, 0,5 to 4 or 5 to 7) in the different questionnaires (Day 0, 7, 14, 30).


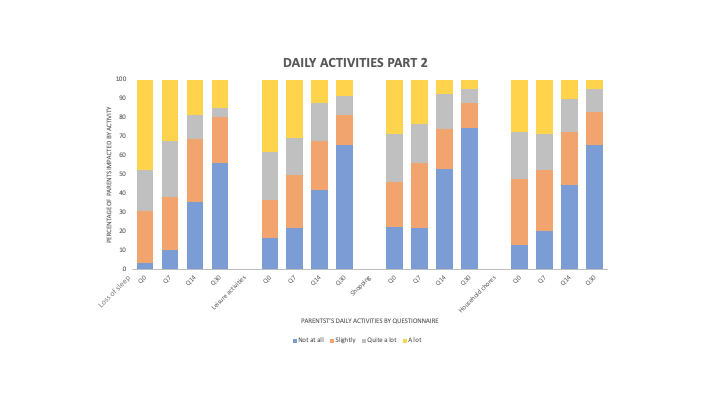


SFigure 7. Dimension 5.n Interference of the child’s disease in the ability to undertake daily activities such as sleeping, leisure activities, shopping or household chores in the four questionnaires (Day 0, 7, 14, 30).

**Annex 5: HRQoL scores by age and dimension**

The following tables shows the health-related quality of life (HRQoL) scores overall and by the different dimensions and age groups evaluated.

| **Health related quality of life score** | | | | | |
| --- | --- | --- | --- | --- | --- |
| Median (IQR) | | | | | |
|  | **Age (months)** | **Q0** | **Q7** | **Q14** | **Q30** |
| **Total** | 0-5 | 0.58  (0.46-0.70) | 0.65  (0.51-0.73) | 0.82  (0.68-0.97) | 0.88  (0.83-1) |
|  | 6-11 | 0.49  (0.43-0. 0.60) | 0.58  (0.47-0.81) | 0.88  (0.62-0.96) | 0.96  (0.68-1) |
|  | 12-23 | 0.45  (0.37-0.64) | 0.61  (0.51-0.92) | 0.77  (0.51-0.92) | 0.96  (0.91-100) |
|  | 0-23 | 0.52  (0.42-0.68) | 0.65  (0.49-0.79) | 0.82  (0.68-0.97) | 0.94  (0.81-1) |

STable 1. Descriptive of the overall health-related quality of life scores for the different age groups. IQR means interquartile range.

| **Health related quality of life score** | | | | | |
| --- | --- | --- | --- | --- | --- |
| Median (IQR) | | | | | |
| **Dimension** | **Age** | **Q0** | **Q7** | **Q14** | **Q30** |
| **Children’s symptoms** | 0-5 months | 70 (59-82) | 79 (64-88) | 93 (84-98) | 96 (88-100) |
|  | 6-11 months | 61 (54-80) | 70 (56-79) | 89 (82-96) | 100 (79-100) |
|  | 12-23 months | 60 (45-75) | 82 (73-91) | 80 (68-96) | 97 (92-100) |
|  | 0-23 months | 70 (55-82) | 77 (63-87) | 91 (82-98) | 96 (88-100) |
| **Children’s**  **behaviour** | 0-5 months | 48 (33-62) | 62 (38-81) | 90 (71-100) | 95 (82-100) |
|  | 6-11 months | 43 (29-52) | 62 (33-76) | 76 (48-90) | 100 (69-100) |
|  | 12-23 months | 45 (21-74) | 57 (57-90) | 76 (48-86) | 100 (93-100) |
|  | 0-23 months | 43 (32-62) | 62 (36-79) | 83 (63-100) | 100 (81-100) |
| **Parent’s concern** | 0-5 months | 57 (38-71) | 71 (52-86) | 90 (81-100) | 95 (86-100) |
|  | 6-11 months | 52 (43-74) | 71 (55-86) | 90 (83-98) | 100 (88-100) |
|  | 12-23 months | 57 (43-67) | 86 (57-90) | 86 (60-98) | 98 (92-100) |
|  | 0-23 months | 57 (43-71) | 71 (52-86) | 90 (81-100) | 95 (86-100) |
| **Parent’s feelings** | 0-5 months | 44 (28-61) | 56 (33-72) | 78 (61-100) | 97 (72-100) |
|  | 6-11 months | 44 (31-56) | 50 (33-78) | 78 (61-100) | 100 (50-100) |
|  | 12-23 months | 50 (40-60) | 67 (44-72) | 75 (49-88) | 100 (75-100) |
|  | 0-23 months | 44 (32-61) | 56 (33-72) | 78 (61-100) | 100(69-100) |
| **Parent’s daily activities** | 0-5 months | 50 (32-70) | 55 (38-68) | 77 (50-100) | 93 (74-100) |
|  | 6-11 months | 48 (28-56) | 55 (25-81) | 86 (50-100) | 100 (66-100) |
|  | 12-23 months | 31 (19-52) | 59. (16-95) | 77 (30-90) | 99 (80-100) |
|  | 0-23 months | 48 (31-66) | 55 (36-75) | 78 (50-100) | 95 (73-100) |

STable 2. Descriptive of health-related quality of life (HRQoL) scores in the five dimensions and for the different age groups.

**Annex 6: Prices used in the healthcare resource consumption estimations**

| **Resource unit** | **Unit cost(€)** |  |
| --- | --- | --- |
| **Consultation of pediatrics and neonatology** | 42,57 |  |
|  |  |  |
| **Stay in pediatrics and neonatology** | 544,08 |  |
| **Specialists visits** | 107,33 |  |
| **Hospital/ambulatory emergency** | 155,89 |  |
| **Stay in ICU** | 1365,29 |  |

Stable 3. Prices for the different settings

| **Pharmaceutical treatment** | **Unit cost(€)** |
| --- | --- |
| **Paracetamol** | 1,75 |
| **Ibuprofen** | 2,5 |
| **Salbutamol** | 4,76 |
| **Budesonide** | 12,41 |
| **Oral steroids** | 4,76 |
| **Antibiotics** | 3,12 |
| **Inhalation chamber** | 7,28 |
| **Inhalation mask** | 11,44 |
| **Physiological serum** | 1,95 |

Stable 4. Prices for the different pharmaceutical treatments
